# An NAM Domain Gene, *GhNAC79*, Improves Resistance to Drought Stress in Upland Cotton

**DOI:** 10.3389/fpls.2017.01657

**Published:** 2017-09-25

**Authors:** Yaning Guo, Chaoyou Pang, Xiaoyun Jia, Qifeng Ma, Lingling Dou, Fengli Zhao, Lijiao Gu, Hengling Wei, Hantao Wang, Shuli Fan, Junji Su, Shuxun Yu

**Affiliations:** ^1^College of Agronomy, Northwest A&F University Yangling, China; ^2^State Key Laboratory of Cotton Biology, Institute of Cotton Research of Chinese Academy of Agricultural Sciences Anyang, China; ^3^School of Life Science, Yulin University Yulin, China

**Keywords:** *GhNAC79*, cotton, stress, drought, development

## Abstract

Plant-specific NAC proteins comprise one of the largest transcription factor families in plants and play important roles in plant development and the stress response. *Gossypium hirsutum* L. is a major source of fiber, but its growth and productivity are limited by many biotic and abiotic stresses. In this study, the NAC domain gene *GhNAC79* was functionally characterized in detail, and according to information about the cotton genome sequences, it was located on scaffold42.1, containing three exons and two introns. Promoter analysis indicated that the *GhNAC79* promoter contained both basic and stress-related elements, and it was especially expressed in the cotyledon of *Arabidopsis.* A transactivation assay in yeast demonstrated that *GhNAC79* was a transcription activator, and its activation domain was located at its C-terminus. The results of qRT-PCR proved that *GhNAC79* was preferentially expressed at later stages of cotyledon and fiber development, and it showed high sensitivity to ethylene and meJA treatments. Overexpression of *GhNAC79* resulted in an early flowering phenotype in *Arabidopsis*, and it also improved drought tolerance in both *Arabidopsis* and cotton. Furthermore, VIGS-induced silencing of *GhNAC79* in cotton led to a drought-sensitive phenotype. In summary, *GhNAC79* positively regulates drought stress, and it also responds to ethylene and meJA treatments, making it a candidate gene for stress studies in cotton.

## Introduction

As a major source of fiber, cotton (*Gossypium hirsutum* L.) shows greater tolerance to drought and salt than wheat and rice, but with the changing of global climate and increasing level of pollution, abiotic stress is becoming a major limiting factor for cotton growth and productivity (Ahuja et al., 2010). Among these factors, high salinity and drought are the main stresses (Wang et al., 2001; Rabbani et al., 2003); currently, more than 10% of arable land is experiencing water shortages, leading to a 50% average reduction in the yield of major crops (Bartels and Sunkar, 2005). Senescence, which consists of a variety of molecular and physiological events, is the last stage of leaf development, and it positively impacts plant reproduction. However, premature senescence negative affects cotton yield and quality. Therefore, improving stress tolerance and delaying leaf senescence in cotton through genetic engineering is a promising production strategy for which candidate genes need to be identified.

NAC proteins are plant-specific transcription factors characterized by a conserved NAC domain (Christianson et al., 2009), whose name was originally derived from the names of proteins containing NAM (no apical meristem), ATAF1/2 and CUC2 (cup-shaped cotyledon). NAM is required for SAM formation during embryogenesis (Souer et al., 1996); ATAF1/2 has dual functions in both abiotic and biotic stress responses (Lu et al., 2006; Jensen et al., 2008); and CUC2 is involved in organ separation (Aida et al., 1997). Recent studies have reported that NAC transcription factors play diverse roles in a variety of stress responses (Fujita et al., 2004; He et al., 2005; Seo et al., 2010) and developmental processes (Xie et al., 2000; Kim et al., 2008), and some are important in the development of roots and floral organs (Ishida et al., 2000; Xie et al., 2000; He et al., 2005), the development of secondary cell walls and lignin (Chai et al., 2015; Xu et al., 2015), fruit ripening and carotenoid accumulation (Xu et al., 2015; Zhou et al., 2015), leaf senescence (Wu et al., 2012; Zhao et al., 2015), and cell death (Wang et al., 2015). Furthermore, some NAC members are involved in the responses to abiotic and biotic stresses, such as drought, salinity, and cold (Li et al., 2014; Wang et al., 2014; Yu X. et al., 2014; Yang et al., 2015), as well as pathogen attack and wounds (Wu et al., 2009; Seo et al., 2010).

Since the publication of the cotton genome sequences (Paterson et al., 2012; Yu J. et al., 2014; Li F. et al., 2015; Zhang et al., 2015), more *NACs* (*GhNACs*) have been studied in detail. There are more than 100 NAC members in cotton, and they have been shown to have a variety of functions (Balazadeh et al., 2010; Kim et al., 2011; Matallana-Ramirez et al., 2013; Qiu et al., 2015) such as in development and in responses to biotic and abiotic stresses (Shah et al., 2013, 2014). In cotton, an *NAC* gene, *GhXND1*, negatively regulates xylem development (Li et al., 2014); *GhNAC12* promotes leaf senescence (Zhao et al., 2016); and *SNAC1* improves drought and salt resistance (Liu et al., 2014). Therefore, the potential functions of *GhNACs* in cotton development and stress responses should be thoroughly studied.

Our study mainly describes an NAC domain gene, *GhNAC79*, that is predominantly expressed in cotyledons and fibers. It promotes flowering in *Arabidopsis* and enhances drought tolerance in both *Arabidopsis* and cotton, and it also responds to drought and different plant hormones, such as ethylene and ABA. This work not only complements previous functional studies of *GhNACs* in cotton but also provides important material for cotton breeding for drought resistance.

## Materials and Methods

### Plant Materials, Growth Conditions and Stress Treatments

The cotton cultivar CCRI10 (premature senescence) was used for gene cloning and subjected to different treatments. Healthy and uniform seeds were selected and cultivated in a culture room at 25°C with a 16-h light/8-h dark cycle. *Arabidopsis thaliana* ecotype Col-0 was used as the parent strain for *Arabidopsis* transformation. Surface-sterilized seeds were sown in 1/2 MS medium and incubated at 4°C for 3 days in dark conditions to break dormancy, and the plants were then cultivated at 22°C with a 16-h light/8-h dark cycle and a relative humidity of approximately 80%.

Cotton was subjected to drought and salinity treatments in three ways. First, detached true leaves were submerged in water for 1 day and then transferred into a 20% PEG6000 or 200 mM NaCl solution, and controls were submerged in water. Samples were collected at 0, 2, 4, 6, 8, 12, and 24 h. Second, roots were submerged in a 20% PEG6000 or 200 mM NaCl solution, and the same amount of water was used for the control. The leaves were collected at 0 Day post anthesis (DPA), 5, 10, 15, and 20 Days. Third, healthy seeds were germinated in sealed glass jars containing either 200 mM mannitol or 200 mM NaCl, and the leaves and roots were collected separately once the seedlings showed an obvious phenotype. The control consisted of only the medium.

Two plant hormone treatments were carried out. First, seedlings at the three-leaf stage were sprayed with 50 μM ABA, 200 μM meJA or 200 μM ethephon (ethephon releases ethylene when dissolved in water), and controls were sprayed with water. The leaf samples were collected at 0, 4, 12, 24, and 48 h after treatment. Second, 25 μM ABA, 100 μM meJA and 100 μM ethephon were individually applied to the sealed glass jars, and then the leaf and root samples were collected separately. The control consisted of only MS medium.

The cotyledons, true leaves, stems, roots and flowers of cotton seedlings were collected for gene expression analyses. Fibers at 0, 5, 10, 15, 20, and 25 DPA were obtained as described previously (Singh et al., 2009). To evaluate the function of *GhNAC79* during cotyledon development, two short-season cotton cultivars, CCRI10 (premature senescence) and Liao4086 (no premature senescence), were selected and cultivated under normal water and nitrogen management. Cotyledon samples were collected every 7 days in each of eight developmental stages from the non-senescent stage to the completely senescent stage. There were three biological replicates for each sample.

### Malondialdehyde (MDA) and Soluble Protein Measurements

The enzymatic solution was prepared as follows: 0.5 g samples were quickly ground in a cold mortar with 8 μl of cold extraction medium (0.05 mol/L, pH = 7.8, Na_2_HPO_4_-NaH_2_PO_4_), and all the liquid was then transferred into a 25 ml centrifuge tube and centrifuged at 20,000 rpm for 30 min at 2°C. The supernatants were collected for further use as the enzymatic solution.

The MDA content was determined according to the method described by Jingqing Zhao et al. (2012) with slight modification. A mixture of 1.5 ml of buffer (0.05 mol/L, pH = 7.8, Na_2_HPO_4_-NaH_2_PO_4_) and 1.5 ml of enzymatic solution was placed in a 10 ml centrifuge tube (the control consisted of 1.5 ml of buffer and 1.5 ml of H_2_O), and 2.5 ml of 0.5% thiobarbituric acid (TBA) was then added to each tube. Finally, the reaction mixture was mixed well, incubated at 100°C for 20 min, and then quickly put on ice. After centrifugation at 1800 *g* for 10 min, the absorbances of the supernatant at 450, 532, and 663 nm were determined with a spectrophotometer. The soluble protein was determined according to the method described by Bradford (1976).

### RNA Extraction, cDNA Synthesis and DNA Preparation

Total RNA was extracted with an RNAprep Pure kit (Tiangen, China), which was suitable for plants rich in polyphenols and amylase. The quality and concentration of the RNA were confirmed by 1% agarose gel electrophoresis and a spectrophotometer, and DNaseI was used to remove the genomic DNA. cDNA synthesis was performed by strictly following the manufacturer’s protocol for ReverTra Ace qPCR RT Master Mix (TOYOBO, Japan). Total genomic DNA was extracted from the cotton leaf tissues by the CTAB method (Song et al., 1998).

### Gene Cloning and Sequence Analysis

The primers used for gene cloning, qRT-PCR, VIGS and promoter cloning were designed with OLIGO7, as shown in **Table 1**. The conserved domain of *GhNAC79* was searched for in NCBI^1^. Using the *G. hirsutum* genome database (Zhang et al., 2015), *GhNAC79* was pitched at scaffold42.1, and the intron and exon structure was analyzed by comparing the genomic and coding sequences. At the same time, an unrooted phylogenetic tree was constructed with 126 AtNACs by MEGA 5.1 (Tamura et al., 2011).

**Table 1:** Primers for gene and promoter cloning, qRT-PCR and VIGS.

Name	Forward primer (5′ → 3′)	Reverse primer (5′ → 3′)	PCR product size (bp)
GhNAC79	AGACTGGCATGAATAAACAAG	AGCTTCTCCATCTACACATCA	759
qRT-PCR-GhNAC79	AATCACATTAATCGGTTTACT	CTAAAGATTCCAAAACCCATC	117
VIGS-GhNAC79	CTACACCTCCTATCCTAAGAC	AGCTTCTCCATCTACACAT	272
PGhNAC79	GTGATGTGTAAAACAGTCTAT	TTCTTCAGTAGGATAGAACCG	1031

*The *GhNAC79* primer was used for gene cloning; the qRT-PCR-GhNAC79 primer was used for fluorescent qRT-PCR; the VIGS-GhNAC79 primer was used for VIGS; and the PGhNAC79 primer was used for promoter cloning.*

### Transcriptional Activation Activity Analysis

According to the domain features of GhNAC79, the entire open reading frame (ORF) was divided into four parts, which were inserted into the pGBKT7 vector with *Eco*RI and *Bam*HI. After sequencing with the T7 universal primer, all the constructed plasmids were transferred into a yeast strain (Y_2_H) by strictly following the Clontech method^2^. The positive yeast strain was selected using SD/-Trp/X-α-gal/25 mM 3-AT medium, and PCR was used to verify the results. The primers were designed based on the sequences of the four fragments and are shown in **Table 2**.

**Table 2:** Primers for the transactivation assay in yeast.

Name	Forward primer (5′ → 3′)	Reverse primer (5′ → 3′)	PCR product size (bp)
ADfra-1	CATGAATAAACAAGTATAGGC	CATGGTCTTGAATAGCTTTAT	462
ADfra-2	CATGAATAAACAAGTATAGGC	TTCTCCCATCTTTAATATCCC	590
ADfra-3	GACCATGTTTCTTTAGCTAAT	CATCTACACATCATCTCTAAA	289
ADfra-4	CATGAATAAACAAGTATAGGC	CATCTACACATCATCTCTAAA	744

### Promoter Cloning and Analysis

For promoter cloning, approximately 1.0 kb sequences of the 5′ UTR were isolated based on the information from the genome database. Using *Xba*I and *Sca*I cutting sites, the promoter was inserted into the pBI121::GUS expression vector and transferred into *Arabidopsis* through *Agrobacterium tumefaciens* strain LBA4404 with GUS as the reporter. At the same time, PlantCARE^3^ was used to predict the important elements in the promoter.

Histochemical staining for GUS activity was conducted by incubating fresh *Arabidopsis* tissues in the GUS staining solution (Jefferson et al., 1987) with the wild type as the control. After one night of incubation at 37°C, the stained tissues were bleached with ethanol ranging from a high concentration to a low concentration until the wild type became white. Tissues were then photographed directly under a stereomicroscope.

### Quantitative RT-PCR (qRT-PCR) Assays

The primer was designed with OLIGO7 according to the C-terminus of the sequence, and the specificity of the primer was ensured by the melting peaks and dissociation curves. The reactions were performed with Go Taq qPCR Master Mix (Promega, United States) on an ABI7500 instrument (Applied Biosystems, United States) with *GhHis3* as the endo-reference gene (Tu et al., 2007). A total reaction volume of 20 μl was used for qRT-PCR as follows: 2 μl of diluted cDNA, 1.0 μl of both the forward and reserve primers, 6 μl of sterile H_2_O, and 10 μl of Go Taq qPCR Master Mix. The running procedure was strictly established as described in the manual, and each collected sample contained three technical replicates. The entire operation was performed under low-light conditions, and the results were calculated using the 2^-ΔΔC_T_^ method (Livak and Schmittgen, 2001), where ΔC_t1_ = Ct_(_*_GhNAC79_*_)_-Ct_(_*_GhHis3_*_)_ and ΔC_t2_ = Ct_1(_*_GhNAC79_*_)_-Ct_1(_*_GhNAC79_*_of control)_. Statistical significance was calculated by analysis of variance (ANOVA) or *t*-tests using SAS software (Knapp et al., 1985).

### Transformation of *Arabidopsis* and Cotton

The *35S::GhNAC79* plasmid was constructed by inserting the coding region into a binary vector, pBI121, with *Xba*I and *Sac*I cutting sites. After sequencing, the *35S::GhNAC79* plasmid was transformed into *A. tumefaciens* strain *LBA4404* by the heat shock method (Bhuiyan et al., 2011), and *Arabidopsis* was transfected with an improved inflorescence-dip method (Clough and Bent, 1998). The T_0_ transgenic plants were germinated on 1/2 MS medium with kanamycin. After 14 days, the green plants were transferred to nutritive soil in a culture room, and PCR was used to analyze the positive plants.

The transformation of cotton was completed at the Institute of Cotton Research of the Chinese Academy of Agricultural Sciences (CAAS). Seeds of CCRI24 (an upland cotton cultivar) were sterilized and germinated on 1/2 MS medium at 28°C in a culture room, and hypocotyls cut from sterile seedlings served as the transformation receptors. The construction of 35S::GhNAC79 was transferred into cotton via *Agrobacterium* (Zhang, 2008). The positive cotton lines were verified with PCR and kanamycin, and the T_1_ generations of the *GhANC79*-overexpression lines were used for further analysis. The primer used for PCR was designed based on the 35S promoter and the *GhNAC79* sequence, as shown in **Supplementary Table S1**.

### Modified Virus-Induced *GhNAC79* Silencing in Cotton

To improve the robustness of the virus-induced cotton *GhNAC79* silencing results, we used two silencing systems: pCLCrVA-pCLCrVB and pYL156-pYL192. Approximately 200 bp of the *GhNAC79* sequence was inserted into the pCLCrVA vector with *Spe*I and *Asc*I cutting sites and pCLCrVB as the helper vector (Gu et al., 2014). pCLCrVA::GhNAC79 and pCLCrVB were transferred into cotton cotyledons through *Agrobacterium* as previously described (Kumagai et al., 1995) with pLCrVA::PDS as the indicator and pLCrVA-infected plants as the negative control. At the same time, 200 bp of the *GhNAC79* sequence was inserted into the pYL156 vector with *Eco*RI and *Bam*HI with pYL192 as the helper vector, pYL156::PDS as the indicator, and pYL156-infected plants as the negative control (Unver and Budak, 2009). The cotton used for infection was cultivated at 22°C until the cotyledons flattened, and the infected cotton seedlings were grown at 22°C under low humidity.

## Results

### Cloning and Characterization of *GhNAC79*

*GhNAC79* (KU963586, unpublished) was cloned from a senescent cotyledon of *G. hirsutum.* According to the genome information, *GhNAC79* was located on scaffold42.1 and contained 3 exons and 2 introns. It further contained a conserved NAM domain and encoded a protein consisting of 231 amino acids with a molecular weight of 26.972 kDa.

**FIGURE 1:**
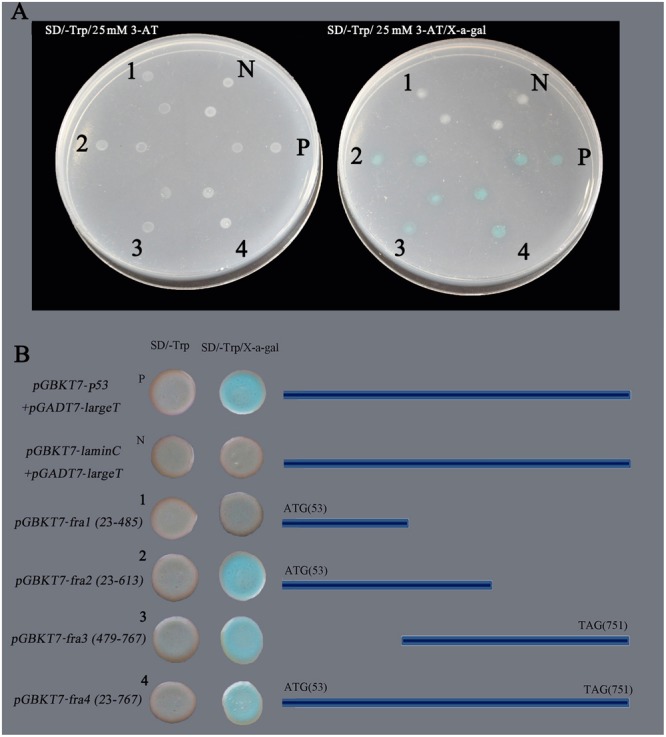
Transcriptional activation activity analysis of *GhNAC79.* The *GhNAC79* sequence was divided into four fragments, which are marked with 1–4 respectively. **(A)** The phenotype of yeast strains growing on SD/-Trp/25 mM 3-AT or SD/-Trp/25 mM 3-AT/x-a-gal media. **(B)** The sequence information for different fragments. N/pGBKT7-LaminC + pGADT7-LargeT: negative control; P/pGBKT7-53 + pGADT7-LargeT: positive control.

The *GhNAC79* sequence was divided into four fragments. The first fragment was from 23 to 485 bp and contained the conserved NAM domain. The second fragment was slightly longer than the first fragment, from 23 to 613 bp, which was selected to verify the definite transcriptional activation domain. The third fragment was from 479 to 767 bp, which avoided the conserved domain. Finally, the fourth fragment contained the complete ORF. The constructed plasmids pGBKT7-fra1, pGBKT7-fra2, pGBKT7-fra3, and pGBKT7-fra4 were transformed into Y_2_H, and the results are shown in **Figure 1**. The positive control stains (pGBKT7-p53 + pGADT7-largeT) appeared blue in color on X-α-Gal substrate, whereas the negative control (pGBKT7-laminC + pGADT7-largeT) exhibited no change in color. The *GhNAC79* fragments, except pGBKT7-fra1, appeared blue in color, which indicated that *GhNAC79* functioned as a transcriptional activator and that its transactivation domain was located at the C-terminus. To ensure the accuracy of the results, all of the stains were affirmed by PCR.

### The Expression Patterns of *GhNAC79* in Special Tissues

**FIGURE 2:**
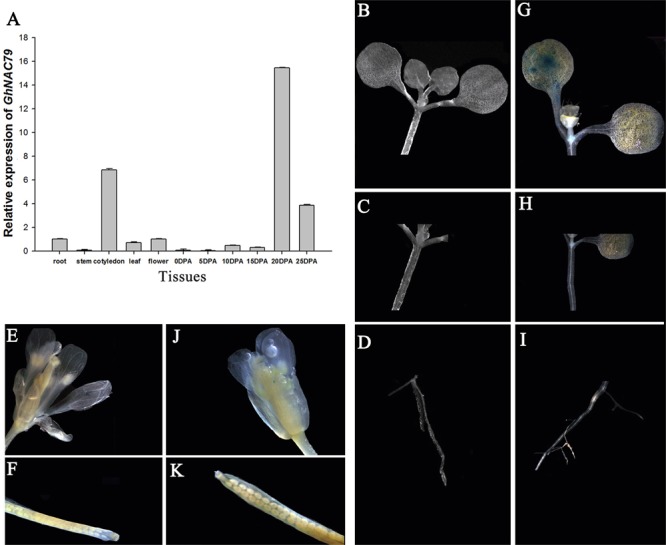
The expression patterns of *GhNAC79* in special tissues. **(A)** Samples of roots, stems, cotyledons, true leaves, flowers, and different developmental fibers were collected, and qRT-PCR was conducted to explore the expression patterns of *GhNAC79*. Data are shown as the mean ± SD (*n* = 3). *GhHIS3* was used as the reference gene. **(B–K)** The *GhNAC79* promoter was transformed into *Arabidopsis* with GUS as the indicator, and photographs were taken under a stereomicroscope. **(B–F)** Different tissues of wild type *Arabidopsis*. **(G–K)** Different tissues of transgenic *Arabidopsis*.

To detect the special expression patterns of *GhNAC79* in different tissues, the roots, cotyledons, stems, true leaves, flowers and fiber at different stages were collected. As shown in **Figure 2A**, *GhNAC79* was mainly expressed in the cotyledon and later stages of fiber development, such as 20 and 25 DPA. In contrast, the expression level was relatively low in the stem and the initial fiber developmental stage.

The *GhNAC79* promoter was cloned, and the sequenced promoter contained parts of the ORF sequences, which demonstrated consistency from the ORF to 5′ UTR. Based on the PlantCARE analysis, the 1.0 kb sequences contained all basal elements, such as the TATA box, CAAT box and 5 UTR Py-rich stretch. Additionally, the promoter contained some stress- and regulation-related *cis*-elements, such as those related to light, circadian rhythm, drought and ethylene, as shown in **Table 3**. The reconstructed vector _promoter_GhNAC79::GUS was transferred into *Arabidopsis*, and histochemical staining was conducted to detect the GUS activity. As shown in **Figures 2B–F**, there was no GUS activity in the wild type, while GUS activity was preferentially detected in the cotyledons and cotyledon bases in transgenic *Arabidopsis*, which is presented in **Figures 2G–K**.

**Table 3:** Stress- and regulation-related cis-elements identified in the *GhNAC79* promoter.

Site name	Species	Position	Sequence	Function
AT1-motif	*Solanum tuberosum*	+42	ATTAATTTTACA	Part of a light-responsive module
Box 4	*Petroselinum crispum*	+42/-648/-782/-893	ATTAAT	Part of a conserved DNA module involved in light responsiveness
HSE	*Brassica oleracea*	+125/+126/+495	AAAAAATTTC	*cis*-acting element involved in heat stress responsiveness
Skn-1_motif	*Oryza sativa*	–172/+262/-453	GTCAT	*cis*-acting regulatory element required for endosperm expression
MBS	*Arabidopsis thaliana*	–232/+615/+916	TAACTG/ CAACTG	MYB-binding site involved in the induction of drought stress
ACE	*Petroselinum crispum*	+465	AAAACGTTTA	*cis*-acting element involved in light responsiveness
GT1-motif	*Avena sativa*	–514	GGTTAAT	Light-responsive element
GT1-motif	*Arabidopsis thaliana*	–515	GGTTAA	Light-responsive element
GARE-motif	*Brassica oleracea*	+626	AAACAGA	Gibberellin-responsive element
circadian	*Lycopersicon esculentum*	+665	CAANNNNATC	*cis*-acting regulatory element involved in circadian control
TCT-motif	*Arabidopsis thaliana*	–701/+840	TCTTAC	Part of a light-responsive element
TC-rich repeats	*Nicotiana tabacum*	+836	GTTTTCTTAC	*cis*-acting element involved in defense and stress responsiveness
GT1-motif	*Solanum tuberosum*	+867	AATCCACA	Light-responsive element
CCAAT-box	*Hordeum vulgare*	–877	CAACGG	MYBHv1-binding site
Sp1	*Zea mays*	+887	CC(G/A)CCC	Light-responsive element
LTR	*Hordeum vulgare*	–1007	CCGAAA	*cis*-acting element involved in low-temperature responsiveness

The promoter of *GhNAC79*-induced GUS was especially expressed in the cotyledon, which was consistent with the expression patterns of *GhNAC79* in cotton tissues. The high expression level of *GhNAC79* in the cotyledon and fiber indicated its special function in these tissues.

### *GhNAC79* Was Also Significantly Upregulated in Later Cotyledon Development Stages

**FIGURE 3:**
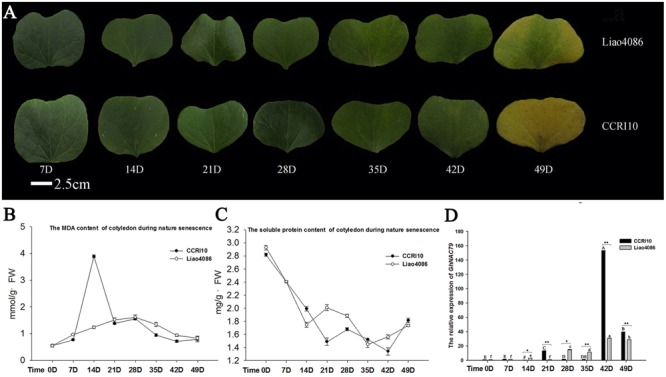
Expression patterns of *GhNAC79* during cotyledon development. **(A)** Seven cotyledon developmental stages. Bar = 2.5 cm. **(B,C)** Changes in MDA and soluble protein contents during cotyledon development. D indicates the number of days after the cotyledon had spread out. **(D)** Expression patterns of *GhNAC79* during cotyledon development. One-way ANOVA was based on varying the times for the two varieties. Different letters indicate a significant difference between two values (*p* < 0.01); capital letters are used for CCRI10 and lowercase for Liao4086. A *t*-test was conducted between two varieties at the same time point. ^∗^Values between two varieties are significantly different at the 0.05 confidence level; ^∗∗^Values between two varieties are significantly different at the 0.01 confidence level. Data are presented as the mean ± SD (*n* = 3). *GhHIS3* was used as the reference gene.

During development, the color of the cotyledon became yellow in both cultivars (**Figure 3A**), and the MDA content sharply increased in CCRI10 but was relatively steady in Liao4086, as shown in **Figure 3B**. As a product of membrane lipid peroxidation, MDA is an important indicator of cell damage (Kramer et al., 1991), and the high level of MDA in CCRI10 indicated premature senility. In terms of soluble protein, the two cultivars did not differ greatly, and the soluble protein contents of both decreased during leaf senescence, as shown in **Figure 3C**.

**Figure 3D** shows that *GhNAC79* was very highly expressed in CCRI10 and Liao4086 in later cotyledon development stages, and the highest changes in expression level were approximately 170-fold in CCRI10 and 40-fold in Liao4086. The expression of *GhNAC79* was significantly higher in CCRI10 than in Liao4086. As CCRI10 was more sensitive to senescence than Liao4086, the higher expression of *GhNAC79* in CCRI10 indicated that it might function in cotyledon senescence.

### *GhNAC79* Was Induced by Abiotic Stresses

**FIGURE 4:**
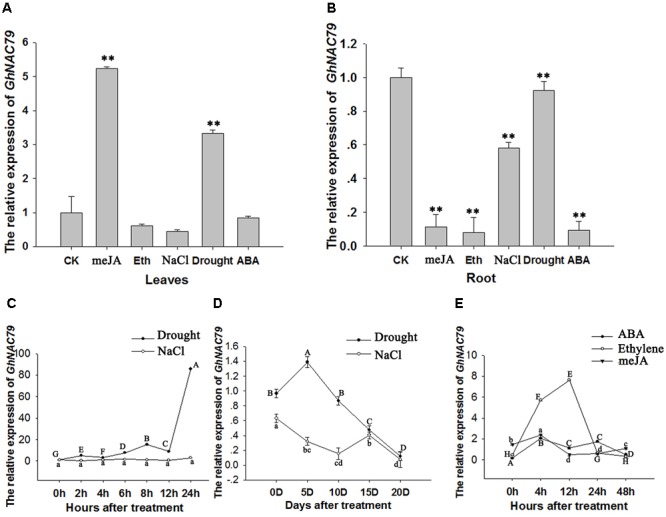
The expression patterns of *GhNAC79* in response to stresses and plant hormones. **(A,B)** Cotton seedlings were cultivated in sealed glass bottles containing MS medium with different treatments. After an obvious phenotype appeared, root and leaf samples were collected separately. **(A)** Expression patterns of *GhNAC79* in response to different stresses and plant hormones in leaves. **(B)** Expression patterns of *GhNAC79* in response to different stresses and plant hormones in roots. **(C,D)** Expression patterns of *GhNAC79* in response to drought and salt treatments. **(C)** Detached leaves were submerged in 20% PEG6000 or 200 mM NaCl, and samples were collected at different time points with h indicating the number of hours after treatment. **(D)** Roots of cotton seedlings were submerged in 20% PEG6000 or 200 mM NaCl, and samples were collected at different time points with D indicating the number of days after treatment. **(E)** Expression patterns of *GhNAC79* in response to ABA, meJA and ethylene treatments with h indicating the number of hours after treatment. Data are presented as the mean ± SD (*n* = 3). *GhHIS3* was used as the reference gene.

To explore the role of *GhNAC79* in response to different stresses, cotton seeds were sown in sealed glass jars containing MS medium subjected to different treatments. As shown in **Figures 4A,B**, *GhNAC79* was induced in leaves by the meJA and drought treatments, but its expression in roots was repressed in all treatments, especially the meJA, ethylene and ABA treatments, by approximately 5∼10 fold. These results indicated that *GhNAC79* might be involved in responses to abiotic stresses.

To verify the response of *GhNAC79* to drought and salt, detached cotton leaves were submerged in 20% PEG6000 or 200 mM NaCl. **Figure 4C** shows that *GhNAC79* was sharply induced at 24 h after drought treatment, whereas *GhNAC79* remained constant under salt treatment. At the same time, after submerging the roots of cotton seedlings in 20% PEG6000 or 200 mM NaCl, *GhNAC79* exhibited an expression pattern indicating sensitivity to drought treatment, as shown in **Figure 4D**. In terms of the response to different plant hormones, *GhNAC79* was very sensitive to ethylene, but its response to ABA and meJA remained relatively steady, as illustrated in **Figure 4E**.

### Overexpression of *GhNAC79* in *Arabidopsis* Resulted in an Early Flowering Phenotype

**FIGURE 5:**
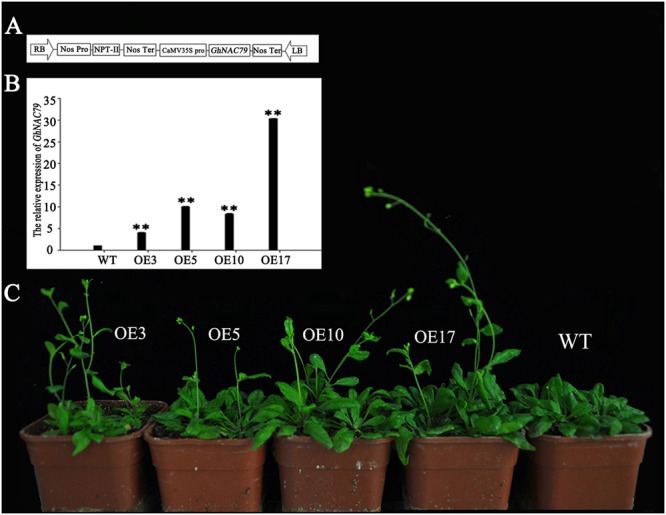
Phenotypes of transgenic *Arabidopsis.* **(A)** Construction of *35S::GhNAC79* vector. **(B)** Expression level of *GhNAC79* in transgenic *Arabidopsis* and wild type. Data are presented as the mean ± SD (*n* = 3) with *GhHIS3* as the reference gene. ^∗∗^Values significantly different from wild type at the 0.01 confidence level. **(C)** An early flowering phenotype of transgenic *Arabidopsis* compared with wild type; Line 3, Line 5, Line 10, and Line 17 were the four lines of transgenic plants.

*35S::GhNAC79* was structured (**Figure 5A**) and transformed into *Arabidopsis* with selective 1/2 MS medium, and the results were verified by PCR. The positive plants were selected and grown in a culture room, and the T_4_-generation transgenic plants were used for phenotype analysis. At the transcriptional level, the expression of *GhNAC79* in the four lines was higher than that of the wild type by approximately 5∼80 fold, as shown in **Figure 5B**. Transgenic *Arabidopsis* also exhibited an early flowering phenotype, which is shown in **Figure 5C**, and the flowering time was approximately 5 days earlier than the wild type, as presented in **Table 4**.

**Table 4:** Comparison of the flowering time for *GhNAC79-*transgenic *Arabidopsis.*

Genotype^a^	Anthesis (DAS)^b^	*N*
Wild type	33.48 ± 1.52	40
OE3	28.35 ± 1.35^∗∗^	40
OE5	29.73 ± 1.73^∗∗^	40
OE10	29.20 ± 1.08^∗∗^	40
OE17	28.78 ± 1.22^∗∗^	40

*Plants were grown under a 16-h light/8-h dark cycle. ^∗∗^Values significantly different from Col-0 at the 0.01 confidence level. ^a^Genetic backgrounds: wild type, and OE3, OE5, OE10, and OE17, which were the four lines of transgenic *Arabidopsis.*^b^Indicators of anthesis (days after sowing) and data are shown as the mean ± standard deviation (SD). *N* represents the number of plants used for analysis.*

### Overexpression of *GhNAC79* in *Arabidopsis* Enhanced Drought Tolerance

**FIGURE 6:**
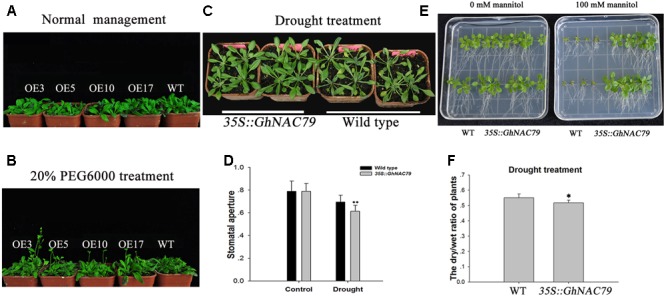
Overexpression of *GhNAC79* in *Arabidopsis* enhanced drought tolerance. **(A,B)** Plants were treated with 20% PEG6000 at 20 days after sowing; wild type represents the wild type, and OE3, OE5, OE10, and OE17 represent the four lines of transgenic *Arabidopsis*. **(A)** Phenotypes of transgenic *Arabidopsis* and wild type under normal management. **(B)** Phenotypes of all plants after drought treatment. **(C,D)** Transgenic plants and wild type were treated with 20% PEG6000 at 14 days after sowing. **(C)** Phenotypes of transgenic plants and wild type. **(D)** Stomatal aperture of transgenic *Arabidopsis* and wild type. **(E)** Phenotypes of transgenic *Arabidopsis* and wild type after treatment with 100 mM mannitol in 1/2 MS medium. **(F)** Differences in dry/wet ratios between transgenic plants and the wild type after mannitol treatment. ^∗^Values significantly different from wild type at the 0.05 confidence level. ^∗∗^Values significantly different from wild type at the 0.01 confidence level.

To explore the function of *GhNAC79* during drought stress, transgenic and wild type *Arabidopsis* were treated with 20% PEG6000 after 20 days of sowing. Under normal water management, all plants reached the vegetable developmental stage, and these results are shown in **Figure 6A**. After drought treatment, transgenic *Arabidopsis* began bolting, but the wild type was still at the vegetable stage, as shown in **Figure 6B**. Additionally, transgenic *Arabidopsis* and wild type were treated with 20% PEG6000 after 14 days of sowing. After 3 days of drought stress, wild type exhibited obvious wilting, while the transgenic *Arabidopsis* remained fresh. Furthermore, the stomatal aperture of transgenic *Arabidopsis* was smaller than that of the wild type, as shown in **Figures 6C,D**.

Transgenic *Arabidopsis* and wild type were germinated in 1/2 MS medium containing 100 mM mannitol, and after approximately 15 days, the transgenic plants displayed an obvious phenotype. As shown in **Figure 6E**, the transgenic plants were stronger than the wild type, and their roots were longer. The dry/wet ratio of the transgenic plants was significantly lower than that of the wild type, as visualized in **Figure 6F**, and the high dry/wet ratio in the wild type implied relatively high water loss. The enhanced drought tolerance in *Arabidopsis* indicated the positive role of *GhNAC79* in drought response.

### *GhNAC79* Played a Positive Role in Drought Stress in Cotton

**FIGURE 7:**
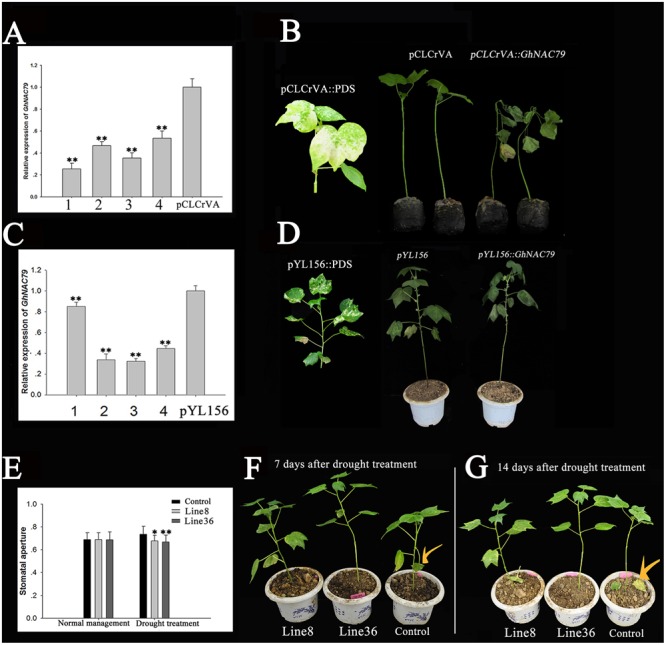
Overexpression of *GhNAC79* enhanced drought tolerance in cotton. **(A,B)** pCLCrVA-pCLCrVB system. **(A)** The expression of *GhNAC79* in virus-infected plants and the control (pCLCrVA). All virus-infected plants were divided into four groups and marked 1–4. **(B)** After drought treatment, the phenotypes of virus-infected cotton seedling and the control with pCLCrVA::PDS as the indicator, pCLCrVA as the control, and pCLCrVA::GhNAC79 representing the virus-infected cotton seedlings. **(C,D)** pYL156-pYL192 system. **(C)** The expression level of *GhNAC79* in virus-infected plants and the control (pYL156). All virus-infected plants were divided into four groups and marked 1–4. **(D)** After drought treatment, the phenotypes of virus-infected cotton seedlings and the control with pYL156::PDS as the indicator, pYL156 as the control, and pYL156::GhNAC79 representing the virus-infected cotton seedlings. **(E–G)** The phenotype of cotton overexpressing *GhNAC79* at the T_1_ stage. Line 8 and Line 36 were two lines of transgenic cotton, and the control was CCRI24 (a cotton cultivar used as a transgenic recipient). **(E)** Stomatal movement of transgenic cotton and control. **(F,G)** After drought treatment, transgenic cotton showed higher drought resistance compared with the control. **(F)** Seven days after drought treatment; **(G)** 14 days after drought treatment. ^∗^Values significantly different from the control at the 0.05 confidence level. ^∗∗^Values significantly different from the control at the 0.01 confidence level. Data are presented as the mean ± SD (*n* = 3). *GhHIS3* was the reference gene.

To study the function of *GhNAC79* in cotton during drought stress, two virus-induced gene silencing (VIGS) systems were used (pCLCrVA-pCLCrVB and pYL156-pYL192). After injection, cotton seedlings were covered with black boxes for one night and then grown in a culture room. As the leaves of the indicator plants exhibited an albino phenotype, the expression level of *GhNAC79* was detected by qRT-PCR. In the pCLCrVA-pCLCrVB system, the expression level of *GhNAC79* was 0.2∼0.6% lower in the infected plants compared with the control (pCLCrVA vector-infected plants), as shown in **Figure 7A**. After treatment with 20% PEG6000, the infected plants showed severe wilting, and all leaves were soft and drooping. However, the young leaves of the control plants remained fresh, as shown in **Figure 7B**. For the pYL156-pYL192 system, the expression of *GhNAC79* decreased by 0.2∼0.9% relative to the control plants (pYL156 vector-infected plants), and after 20% PEG6000 treatment, the infected plants showed more wilting than the control, as shown in **Figures 7C,D**.

To analyze the overexpression of *GhNAC79* in different cotton lines, the roots of two transgenic cotton lines and a wild type (CCRI24) were submerged in 20% PEG6000, and the results are shown in **Figures 7E–G**. After 7 days of treatment, the leaves of the wild type appeared wilted or had dropped, while the transgenic cotton showed normal growth. After 14 days, the wild type had dropped three true leaves, but the two transgenic lines had dropped one leaf and one cotyledon and had another unhealthy cotyledon. Furthermore, there was no significant difference in stoma number and the size of the stomatal aperture between the transgenic lines and the wild type under normal water management, but after drought treatment, the stomatal apertures were smaller in the two transgenic lines compared to the wild type.

Two VIGS-induced *GhNAC79* silencing systems resulted in the cotton being more sensitive to drought, while overexpression of *GhNAC79* increased the drought tolerance of cotton, which demonstrated the positive role of *GhNAC79* in drought tolerance in cotton.

## Discussion

A phylogenetic tree was built with 126 AtNACs and GhNAC79 protein sequences, as shown in **Supplementary Figure S1**. *GhNAC79* belongs to the NAM subfamily, many members of which are involved in plant development; for example, *AT2G17040.1/ANAC036* are associated with a dwarf phenotype and distorted leaves in *Arabidopsis* (Kato et al., 2010). Overexpression of *GhNAC79* in *Arabidopsis* promotes flowering, which enriches the function of NACs in cotton development. Four homologous genes were obtained with three CDs genomes in cotton (A, D and AD), and a multiple alignment was created by DNAMAN software, as shown in **Supplementary Figure S2**. *GhNAC79* differs from *CotAD_11909* by only a single base (a gene from *G. hirsutum* may have originated from *G. arboreum*), and this single-base difference may have been caused by a sequencing error or cultivar variation.

Overexpression of *GhNAC79* in *Arabidopsis* leads to an early flowering phenotype, indicating that *GhNAC79* promotes flowering. *GhNAC79* also exhibits special expression patterns in three differently maturing cotton varieties, as shown in **Supplementary Figure S3**, CCRI74 (early maturing) > Shan70 (middle-maturing) > Bo1 (late-maturing), which further indicate the potential function of *GhNAC79* in flowering. The fiber of *G. arboreum* is spinnable (Xu et al., 2010), but that of *G. raimondii* is not. *GhNAC79* is predominantly expressed in later fiber development stages and comes from the A-subgenome, which implies a potential role of *GhNAC79* in fiber development. The high sensitivity to ethylene exhibited by *GhNAC79* indicates that ethylene plays important roles in fiber elongation (Shi et al., 2006; Qin et al., 2007), so we compare the fiber length between CCRI24 and 35S-GhNAC79 plants at T_2_ stage. As shown in **Supplementary Figure S4**, the length of 35S-GhNAC79 plants is longer than CCRI24, but not significant, but there need more data in different years and areas to support this result.

**FIGURE 8:**
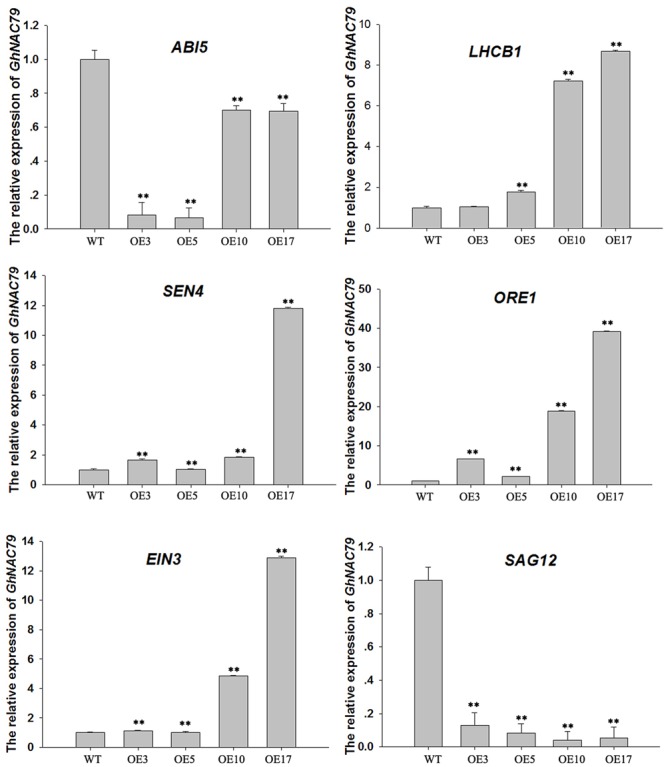
Expression levels of 6 senescence-related genes in transgenic and wild type *Arabidopsis.* ^∗∗^Values significantly different from wild type at the 0.01 confidence level. Data are presented as the mean ± SD (*n* = 3). *GhHIS3* was used as the reference gene.

Additionally, we collected half-yellow rosette leaves from transgenic and wild type *Arabidopsis* to assess the expression of some senescence-related genes with qRT-PCR, and the results are shown in **Figure 8**. *ORE1* coordinates with *EIN3* to regulate leaf senescence through ethylene (Qiu et al., 2015), and the expressions levels of *ORE1* and *EIN3* were higher in the four transgenic lines than in wild type. Because *GhNAC79* in cotton is very sensitive to ethylene treatment, its promoter contains an ethylene-related element, and its overexpression in *Arabidopsis* resulted in an early bolting phenotype under ethylene treatment (**Supplementary Figure S5**). Therefore, *GhNAC79* may play some roles in the ethylene pathway. ABA plays a very important role in drought tolerance (Belimov et al., 2014; Osakabe et al., 2014; Shinohara and Leskovar, 2014) and regulates growth after germination through *ABI5* (Lopez-Molina et al., 2002; Albertos et al., 2015). The expression of *ABI5* in transgenic lines is lower than in the wild type, and overexpression of *GhNAC79* enhanced the tolerance of *Arabidopsis* to ABA treatment, which illustrates the vital role of *GhNAC79* in the ABA pathway. *LhCB1* functions in state transitions during light harvesting in *Arabidopsis* photosynthesis (Pietrzykowska et al., 2014), and photosynthetic ability is an important indicator of leaf senescence. In contrast, *SAG12* is a negative marker gene for leaf senescence (Chang et al., 2003; Merewitz et al., 2011). *LhCB1* had a relatively high expression level in the transgenic lines, while the expression level of *SAG12* was low, which indicated that senility was delayed in the transgenic lines. *SEN4* has a positive influence on plant survival as its homologous gene is related to DNA damage (Takeda et al., 2004), and *SEN4* expression was relatively high in transgenic plants, which indicated the role of *GhNAC79* in plant development. The expression levels of *ABI5, LhCB1, SAG12*, and *SEN4* implied that *GhNAC79* may negatively regulate leaf senescence in *Arabidopsis*. However, high levels of *ORE1* and *EIN3* promoted leaf senescence, and this contradiction may be due to the complexity of leaf senescence.

It is interesting that *GhNAC79* was induced in leaves by meJA and drought treatments, but the expression of *GhNAC79* in roots was repressed in all treatments. For this result, it may caused by the experiment method we used. As all treatments applied in medium, when phenotype appearing in leaf, the condition of roots is not good. Bad condition of roots may be related with low level of *GhNAC79*, so *GhNAC79* may a positive regulator in stress responses. In the same time, drought, Eth and ABA treatments all results in an early bolting phenotype in transgenic *Arabidopsis*, and we think it caused by stress reaction of plant. For plants, they will try their best to accomplish reproduction, when stress coming, through earlier blooming, they get seeds, which make their life continuing.

Under open and sealed conditions, drought stress sharply induced *GhNAC79* in leaves. Overexpression of *GhNAC79* enhanced drought tolerance in *Arabidopsis* and cotton, and *GhNAC79* repression made cotton more sensitive to drought, which demonstrates its potential function in drought stress. Drought is always associated with ABA (Li C. et al., 2015; Tombesi et al., 2015; Yin et al., 2015), so we explored the relationship between *GhNAC79* and ABA. **Supplementary Figure S6A** shows that transgenic *Arabidopsis* exhibited an early bolting phenotype after 15 days of ABA treatment, and wild type growth was obviously inhibited. ABA regulates drought stress through stomatal movements (Cai et al., 2015; Cohen et al., 2015), so the number and aperture size of the stomas were measured after 3 days of ABA treatment. As shown in **Supplementary Figure S6B**, the stomatal aperture decreased after ABA treatment, but the average stomatal aperture was smaller in transgenic *Arabidopsis*. Therefore, we conclude that *GhNAC79* may regulate drought stress in an ABA-dependent manner in *Arabidopsis*. Upland cotton is allotetraploid, and the internal mechanism underlying drought signal transmittance is complex. The expression of *GhNAC79* in leaves was not strongly affected by ABA treatment, but under sealed conditions, the *GhNAC79* in the roots was greatly altered by ABA treatment. Therefore, the repression of *GhNAC79* makes cotton more sensitive to drought stress, whereas overexpression enhances drought tolerance. Furthermore, *GhNAC79* is highly expressed in drought-resistant cotton varieties after drought treatment (**Supplementary Figure S7**). The promoter of *GhNAC79* contains a drought-related *cis*-element, so we consider *GhNAC79* to be a drought-response gene.

## Conclusion

This study proves that *GhNAC79* promotes flowering in *Arabidopsis*, and it also acts as a positive regulator during drought stress through stomatal movement and may be involved in an ABA signal-related pathway. At the same time, *GhNAC79* appears to be involved in the ethylene signal pathway, and because its promoter contains an ethylene-related *cis*-element, its expression is highly induced by ethylene, and its overexpression makes *Arabidopsis* more sensitive to ethylene. Interestingly, *GhNAC79* comes from the A-genome, which is preferentially expressed in later fiber development, and it shows high sensitivity to ethylene treatment, indicating the preferential role of *GhNAC79* in fiber elongation. However, confirming this role requires additional evidence.

## Author Contributions

SY, SF, HlW, and CP designed the experiments. LD and LG collected the sequences, YG performed the experiments and wrote the manuscript, HtW, XJ, JS, and QM revised the language. All the authors read and approved the final manuscript.

## Conflict of Interest Statement

The authors declare that the research was conducted in the absence of any commercial or financial relationships that could be construed as a potential conflict of interest.

## Footnotes

1 http://www.ncbi.nlm.nih.gov/Structure/cdd/wrpsb.cgi

2 http://www.clontech.com/xxclt_searchResults.jsp

3 http://bioinformatics.psb.ugent.be/webtools/plantcare/html/

## Supplementary Material

FIGURE S1 Phylogenetic relationships among GhNAC79 and 126 AtNACs. Multiple alignments of GhNAC79 and AtNACs were executed using Clustal X, and the phylogenetic tree was constructed using MEGA 5.1 and the neighbor-joining (NJ) method.

FIGURE S2 Sequence alignments of *GhNAC79* and its predicted homologous genes in A, D and AD genomes.

FIGURE S3 Expression patterns of *GhNAC79* in the apical bud of three varieties. CCRI74: an early maturing variety; Shan70: a middle-maturity variety; Bo1: a late-maturing variety. Three-leaf to 6-leaf represent the different developmental stages of cotton.

FIGURE S4 The comparison of fiber length between CCRI24 (wild type) and 35S-GhNAC79 (transgenic cotton).

FIGURE S5 Phenotypes of transgenic and wild type *Arabidopsis* after different treatments.

FIGURE S6 Phenotypes of transgenic and wild type *Arabidopsis* after ABA treatment. **(A)** Plants were treated with ABA. **(B)** Stomatal aperture of transgenic and wild type *Arabidopsis* after ABA treatment. Control: transgenic and wild type plants were treated with water.

FIGURE S7 The expression levels of *GhNAC79* after drought treatment in different drought-resistant cotton varieties. ZhongjianD228 and ZhongH177: two drought-resistant cotton varieties. ZhongS9612 and Zhongjian9648: two drought-sensitive cotton varieties. Data are presented as the mean ± SD (*n* = 3). ^∗^: Values significantly different from wild type at the 0.05 confidence level, ^∗∗^: Values significantly different from wild type at the 0.01 confidence level. *GhHIS3* was the reference gene.

TABLE S1 Primer used for selection of transgenic *Arabidopsis* and cotton.
