# Supplementary figures and images for: An NAM Domain Gene, GhNAC79, Improves Resistance to Drought Stress in Upland Cotton
Source: Front Plant Sci. 2017 Sep 25;8:1657. doi: 10.3389/fpls.2017.01657 (PMC5622203; doi:10.3389/fpls.2017.01657)

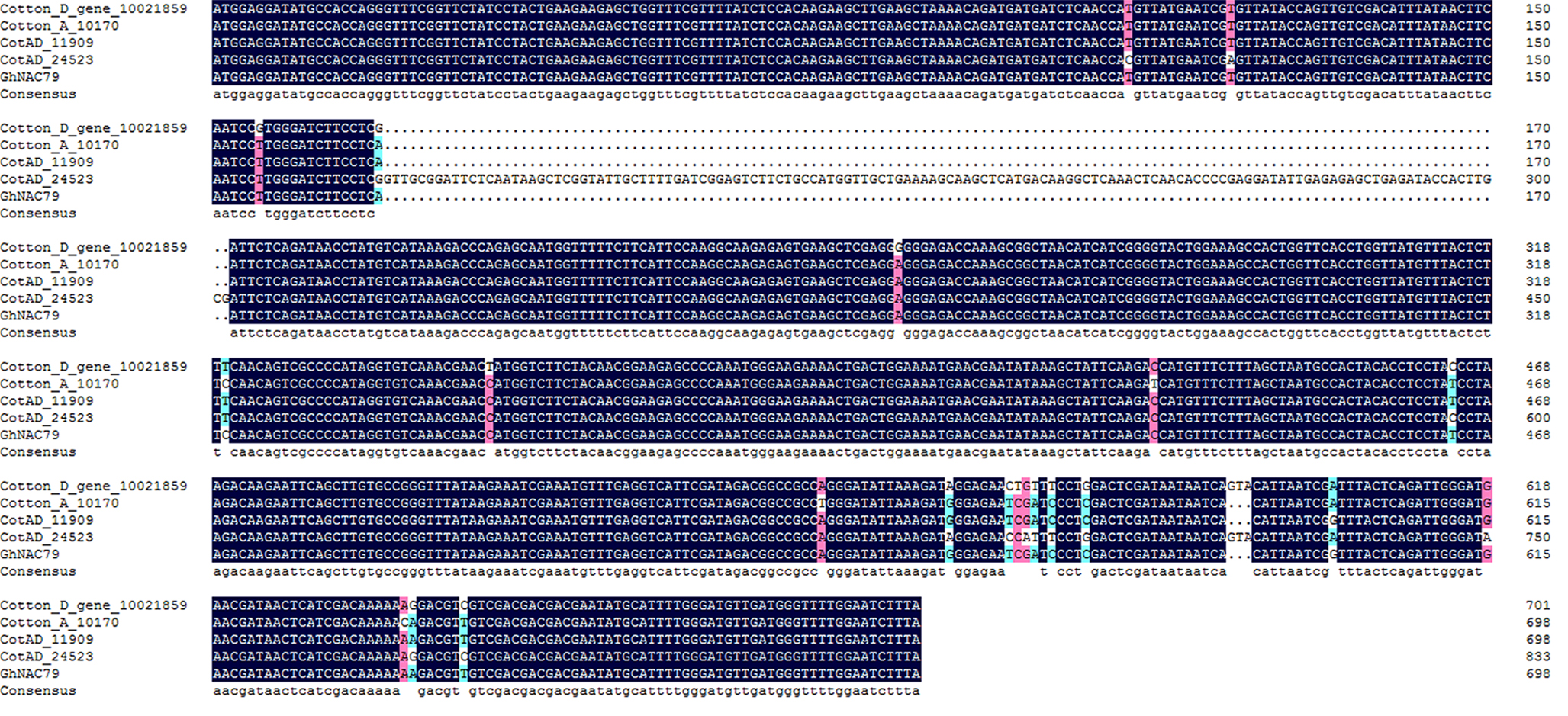

Supplement: FIGURE S2 — Sequence alignments of GhNAC79 and its predicted homologous genes in A, D and AD genomes. [file Image_2.JPEG]

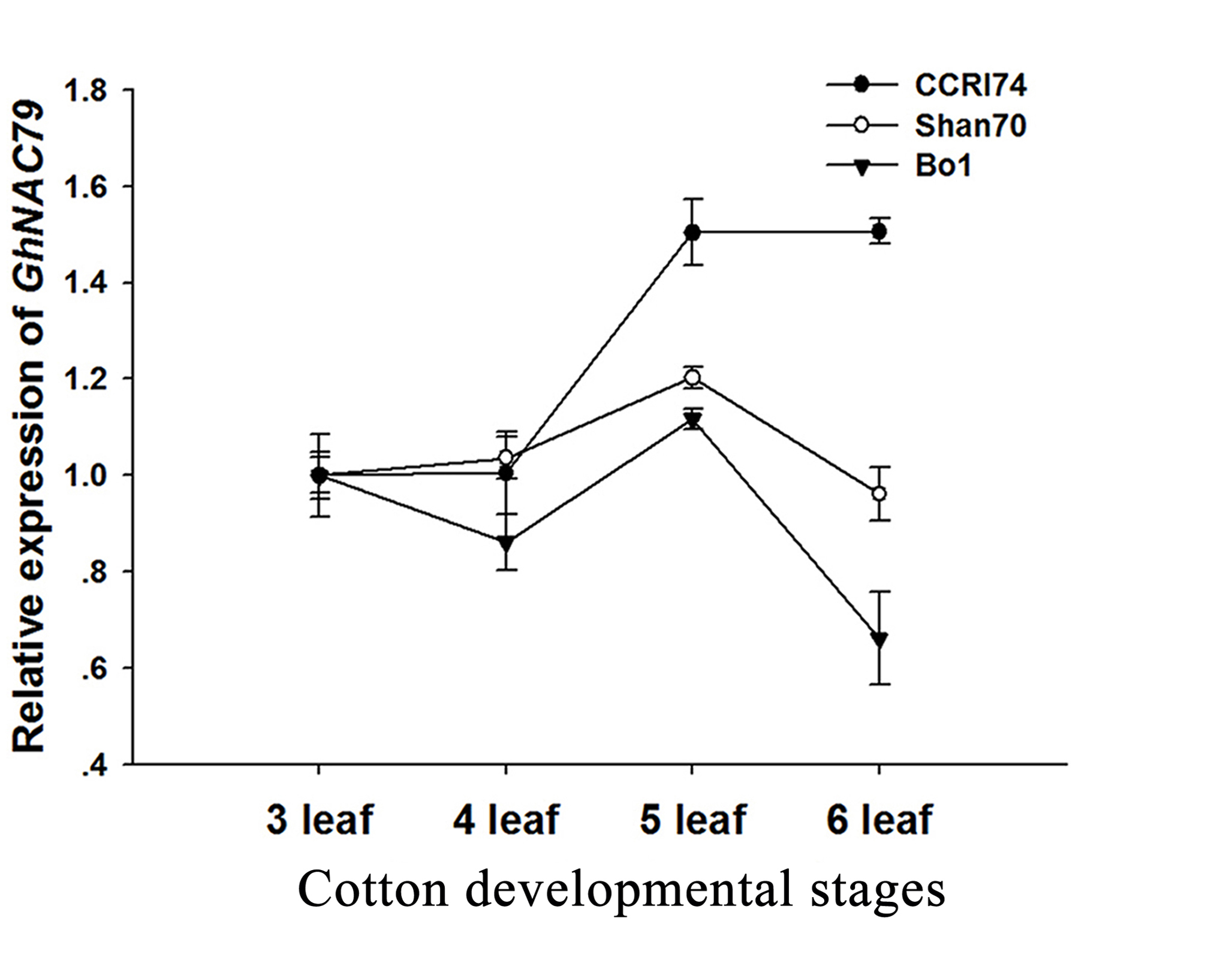

Supplement: FIGURE S3 — Expression patterns of GhNAC79 in the apical bud of three varieties. CCRI74: an early maturing variety; Shan70: a middle-maturity variety; Bo1: a late-maturing variety. Three-leaf to 6-leaf represent the different developmental stages of cotton. [file Image_3.JPEG]

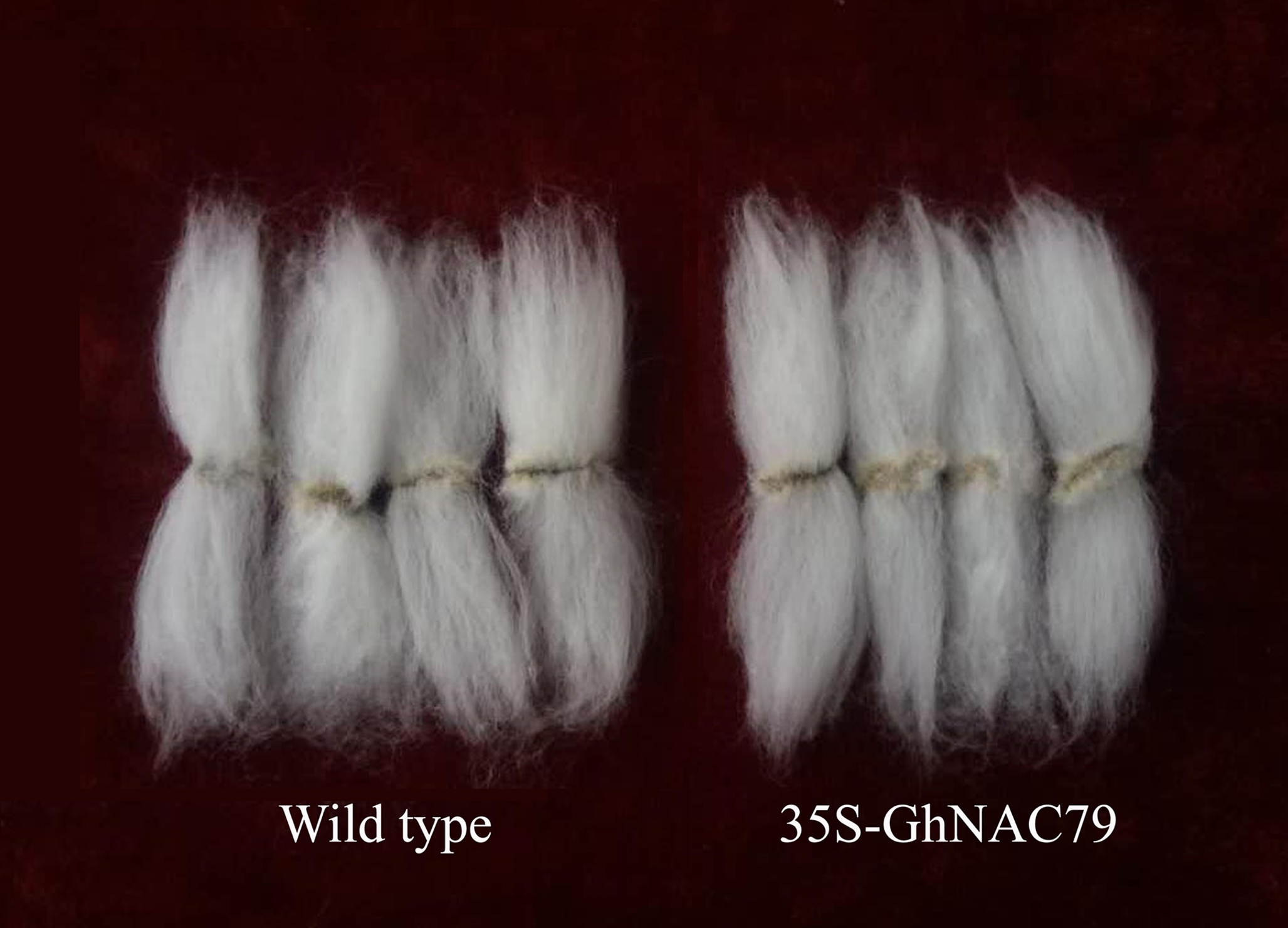

Supplement: FIGURE S4 — The comparison of fiber length between CCRI24 (wild type) and 35S-GhNAC79 (transgenic cotton). [file Image_4.JPEG]

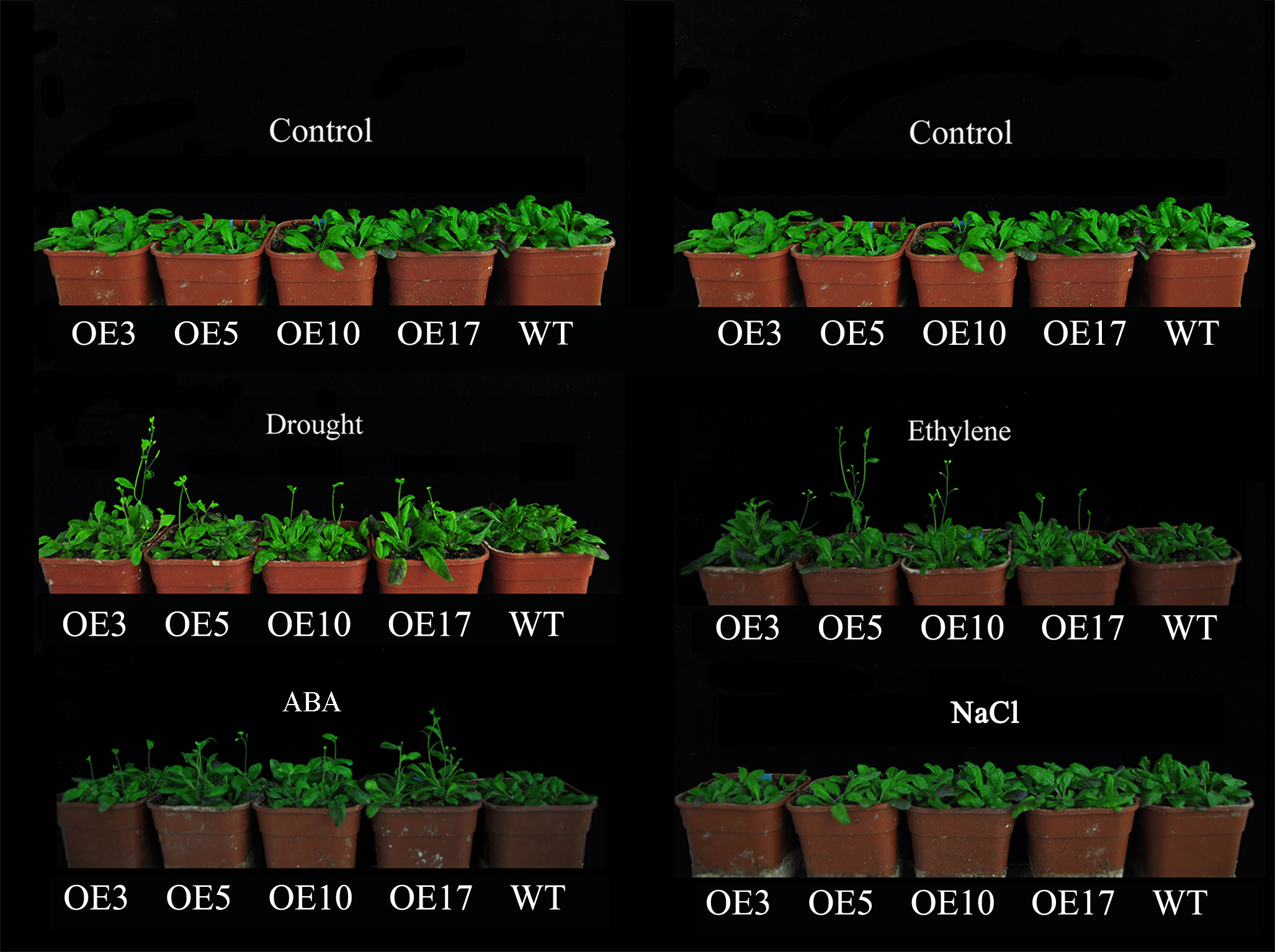

Supplement: FIGURE S5 — Phenotypes of transgenic and wild type Arabidopsis after different treatments. [file Image_5.JPEG]

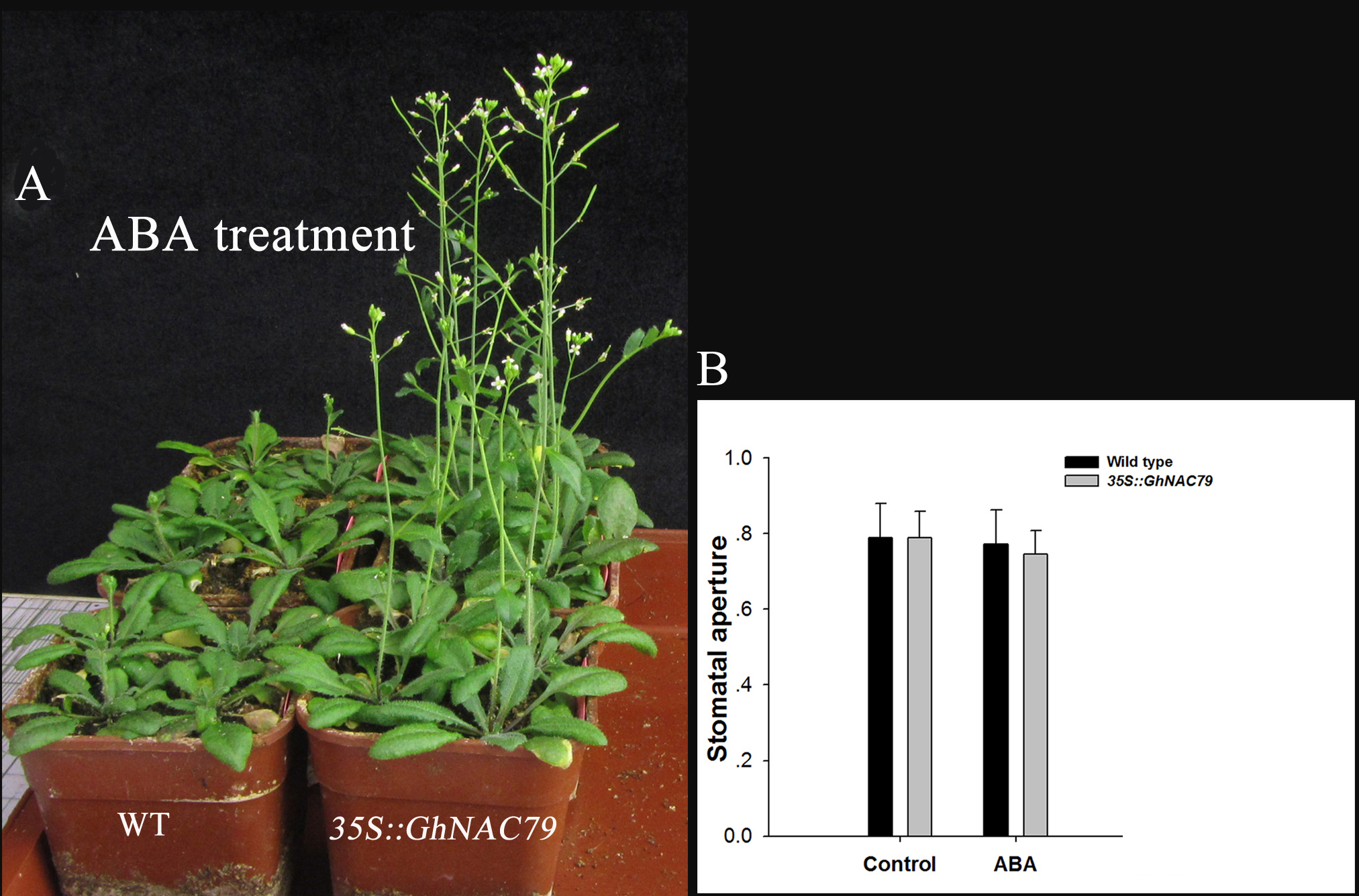

Supplement: FIGURE S6 — Phenotypes of transgenic and wild type Arabidopsis after ABA treatment. (A) Plants were treated with ABA. (B) Stomatal aperture of transgenic and wild type Arabidopsis after ABA treatment. Control: transgenic and wild type plants were treated with water. [file Image_6.JPEG]

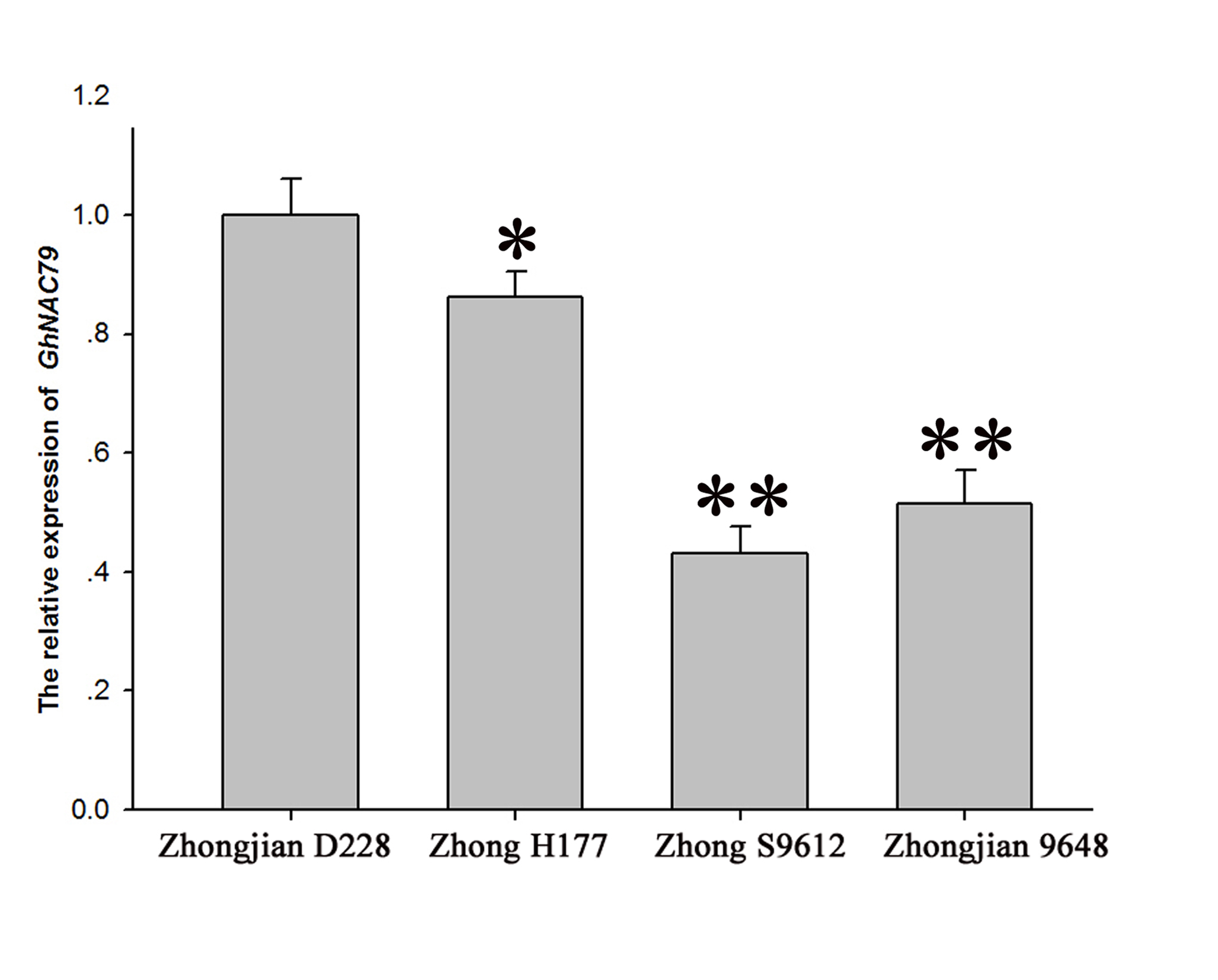

Supplement: FIGURE S7 — The expression levels of GhNAC79 after drought treatment in different drought-resistant cotton varieties. ZhongjianD228 and ZhongH177: two drought-resistant cotton varieties. ZhongS9612 and Zhongjian9648: two drought-sensitive cotton varieties. Data are presented as the mean ± SD (n = 3). ∗: Values significantly different from wild type at the 0.05 confidence level, ∗∗: Values significantly different from wild type at the 0.01 confidence level. GhHIS3 was the reference gene. [file Image_7.JPEG]
