# Supplementary material for: An NAM Domain Gene, GhNAC79, Improves Resistance to Drought Stress in Upland Cotton
Source: Front Plant Sci. 2017 Sep 25;8:1657. doi: 10.3389/fpls.2017.01657 (PMC5622203; doi:10.3389/fpls.2017.01657)
Supplement: TABLE S1 — Primer used for selection of transgenic Arabidopsis and cotton. [file Table_1.docx]

**Supplementary Table 1**

| Name | Forward primer | Reverse primer | PCR product |
| --- | --- | --- | --- |
|  | (5′→3′) | (5′→3′) | size (bp) |
| Check | CCTAACAGAACTCGCCGTA | TCCCAATCTGAGTAAACCGAT | 1.0 kb |
